# Metabolic Profile and Inflammatory Responses in Dairy Cows with Left Displaced Abomasum Kept under Small-Scaled Farm Conditions

**DOI:** 10.3390/ani5040396

**Published:** 2015-10-13

**Authors:** Fenja Klevenhusen, Elke Humer, Barbara Metzler-Zebeli, Leopold Podstatzky-Lichtenstein, Thomas Wittek, Qendrim Zebeli

**Affiliations:** 1 Department of Farm Animals and Veterinary Public Health, Institute of Animal Nutrition and Functional Plant Compounds, Vetmeduni Vienna, Veterinaerplatz 1, Vienna 1210, Austria; E-Mails: fenjaklevenhusen@aol.com (F.K.); elke.humer@vetmeduni.ac.at (E.H.); 2 Department of Farm Animals and Veterinary Public Health, University Clinic for Swine, Vetmeduni Vienna, Veterinaerplatz 1, Vienna 1210, Austria; E-Mail: barbara.metzler@vetmeduni.ac.at; 3 Veterinary Clinic Dr. Leopold Podstatzky-Lichtenstein, Wels 4600, Austria; E-Mail: leopold.podstatzky@raumberg-gumpenstein.at; 4 Department of Farm Animals and Veterinary Public Health, University Clinic for Ruminants, Vetmeduni Vienna, Veterinaerplatz 1, 1210 Vienna, Austria; E-Mail: thomas.wittek@vetmeduni.ac.at

**Keywords:** metabolic disease, acute phase response, periparturient dairy cow, milk yield, cow welfare

## Abstract

**Simple Summary:**

This research established an association between lactation number and milk production and metabolic and inflammatory responses in high-producing dairy cows affected by left abomasal displacement in small-scaled dairy farms. The study showed metabolic alterations, liver damage, and inflammation in the sick cows, which were further exacerbated with increasing lactation number and milk yield of the cows.

**Abstract:**

Left displaced abomasum (LDA) is a severe metabolic disease of cattle with a strong negative impact on production efficiency of dairy farms. Metabolic and inflammatory alterations associated with this disease have been reported in earlier studies, conducted mostly in large dairy farms. This research aimed to: (1) evaluate metabolic and inflammatory responses in dairy cows affected by LDA in small-scaled dairy farms; and (2) establish an association between lactation number and milk production with the outcome of metabolic variables. The cows with LDA had lower serum calcium (Ca), but greater concentrations of non-esterified fatty acids (NEFA) and beta-hydroxy-butyrate (BHBA), in particular when lactation number was >2. Cows with LDA showed elevated levels of aspartate aminotransferase, glutamate dehydrogenase, and serum amyloid A (SAA), regardless of lactation number. In addition, this study revealed strong associations between milk yield and the alteration of metabolic profile but not with inflammation in the sick cows. Results indicate metabolic alterations, liver damage, and inflammation in LDA cows kept under small-scale farm conditions. Furthermore, the data suggest exacerbation of metabolic profile and Ca metabolism but not of inflammation and liver health with increasing lactation number and milk yield in cows affected by LDA.

## 1. Introduction

The transition period (*i.e.*, the period occurring two weeks prepartum through two to four weeks postpartum) is marked by extensive dietary, metabolic, endocrine, and immunological changes in dairy cows [1,2]. This period is also characterized by a high incidence of metabolic diseases, including left displaced abomasum (LDA) in post-partum dairy cows [3]. Displaced abomasum is a severe disease that affects between 0.8% and 6.3% of dairy cows, mostly within 4 weeks postpartum [4]. This disease is characterized by varying degrees of abomasal distension and displacement [5] and is preferably treated by surgical correction. Displaced abomasum has strong negative impacts on overall production efficiency of the afflicted farms mainly due to high related costs (approximately $494 per case) [6], and direct association to decreased herd health status and fertility [5,7].

Although the mechanisms behind LDA are not completely known, research has reported significant associations between the incidence of LDA and a negative energy balance (NEB) prepartum, often reflected in elevated circulating non-esterified fatty acids (NEFA**)** and beta-hydroxy-butyrate (BHBA) concentrations [1,8]. Other predisposing factors for LDA seem to include the first four weeks of lactation [9], genetics [10,11], neurological disorders linked to hypomotility/atony of the abomasum [9,12], metabolic disorders (e.g., ketosis, off-feed), as well as other concomitant diseases, such as endometritis, mastitis, and claw disorders [9], which lead to activation of the acute phase response (APR), characterized by elevated levels of blood acute phase proteins (APP) in the affected cows [12]. Indeed, multiple studies have reported increased blood concentrations of the APP haptoglobin (Hp) and serum amyloid A (SAA) in cows with LDA [11,12,13,14,15].

Despite this large volume of research around LDA, there is a paucity of information, in particular, with regards to potential metabolic and inflammatory conditions associating LDA in small-scale dairy farms. As shown by Stengärde *et al.* [16] the incidence of LDA increases with the size of the herd. Small-scale farms have fewer cows and more individual management options than medium or large size farms [16,17], possibly paying more attention to individual cows. On the other hand, the small-sized farms may have less access to modern technology, likely impairing their capability to properly manage LDA.

The role of lactation number and of milk production in the incidence of LDA and, most importantly, in metabolic and APR variables has not yet been clarified. For example, Cameron *et al.* [8] observed a decreased incidence rate of LDA when lactation number increased, but other field studies have reported an opposite trend [18], or no significant effect of the lactation number on the frequency of LDA [19]. Contradictory observations have also been reported on the relationship of milk yield and LDA [9], and no data are available to which extent these factors are able to modulate metabolic and APR responses. Therefore, this research aimed to evaluate metabolic and APR responses in high-producing dairy cows affected by LDA in small-scale dairy farms additionally taking into account the lactation number and milk production of LDA cows as compared to their healthy counterparts.

## 2. Material and Methods

### 2.1. Animals and Treatments 

Fourteen small-scaled dairy farms in Austria having between 14 to 88 cows/farm (median value: 55 cows/farm) were included in this longitudinal study. All of them were enrolled in the milk recording program of the Association of Austrian Cattle Breeders. All experimental procedures were in compliance with the institutional guidelines and national legislation for animal use in research and approved by the institutional animal care committee. All owners gave informed consent for cows to be included in this study and undergo the testing procedures. Complete information regarding farm conditions, health records, and milk production and composition of the cows was recovered. Average milk production of farms involved in the study ranged from 7765 to 12,073 kg/cow/year with 3.8% to 4.5% fat, and 3.2% to 3.6% protein in the milk. From a total of 20 dairy cows from these farms with LDA blood samples were taken. All farms had a free stalls system and feeding consisted of partial mixed ration and supplementary concentrate mixture for dry and fresh cows during close-up and early postpartum periods, respectively.

Samples of other 20 postparturient clinically-healthy dairy cows of possibly close lactation stage, number, and breed with the LDA cows were used to contrast the results of serum analyses of cows with LDA. In the LDA group, 16 cows were Holstein Friesian and four cows were Simmental breed. The LDA cows had on average 2.9 lactations (four cows in first lactation, six cows in second lactation, three cows in third lactation, four cows in the fourth lactation, and one cow in the fifth, sixth, and seventh lactation each). Cows of the control group (17 Holstein cows and three Simmental cows) had on average of 2.5 lactations (two cows in first lactation, nine cows in second lactation, seven cows in third lactation, and two cows in the fourth lactation). To evaluate the effect of cow’s lactation number on metabolic variables in cows affected with LDA or not, cows were classified in two different lactation groups: cows with ≤2 lactations (*n* = 10 in LDA group and *n* = 11 in the control group) or cows with >2 lactations (*n* = 10 in LDA group and *n* = 9 in the control group).

Cows were diagnosed with LDA from day 1 to 30 (on average: 14 d) postpartum and were immediately submitted to surgery. The clinical diagnose of LDA was confirmed by this surgery intervention via right-flank laparotomy under local analgesia (Procamidor 20 mg/mL, Richter Pharma AG, Wels, Austria). 25 mL were injected twice subcutaneously and intramuscularly in the incision line. The abomasal body was displaced to the left dorsal abdominal quadrant between the left body wall and the rumen. After abdominal exploration and confirmation of LDA, the abomasum was decompressed, repositioned and fixated by omentopexy to its normal anatomical position. All cows underwent surgical correction of LDA while standing. To ensure a rapid and smooth recovery from the surgery, the cows affected with LDA were administered 50 mL of a combination of penicillin and streptomycin intramuscularly (Pen Strep, Univet, Irland) and intravenous infusion of 500 mL glucose solution (Glucose B. Braun 200 mg/mL Infusionslösung, B. Braun Melsungen AG, Melsungen, Germany). All cows that underwent LDA surgery recovered fully.

### 2.2. Blood Sampling

Blood samples were collected from the coccygeal vein shortly prior to surgery for the LDA cows. Blood samples from healthy cows were collected on average 16 ± 5.2 d (mean ± SD) postpartum, a sampling time being comparable with 14 ± 8.9 d postpartum for LDA cows. Serum 10 mL vacutainer tubes (Vacuette, Greiner bio-one, Kremsmünster, Austria) were used to collect blood samples. After collection, blood samples were shortly allowed to clot and serum was separated by centrifuging at 3000× *g* at 4 °C for 20 min. Serum samples were stored at −20 °C until analysis.

### 2.3. Serum Analyses

Concentrations of SAA and Hp in serum were determined by commercially-available ELISA kits according to the methods described previously [15,20]. In brief, serum samples for SAA were initially diluted 1:500, and samples with optical density values above the range of the standard curve were diluted further (1:400 or 1:250) and re-analyzed. No dilution of serum was necessary for measurement of Hp. All samples were tested in duplicate and the optical density values were read on the iMark microplate absorbance reader (Bio-Rad Laboratories GmbH, Vienna, Austria) at 450 nm. The intra-assay variation of all APP assays was controlled by coefficient of variations limits ≤10%.

Serum concentrations of BHBA, NEFA, Calcium (Ca), Phosphorus (P), blood urea nitrogen (BUN), cholesterol, and liver enzymes such as aspartate aminotransferase (AST), glutamate dehydrogenase (GLDH) and gamma-glutamyltransferase (GGT) were measured at the platform for laboratory diagnostics of the University of Veterinary Medicine, Vienna, using standard enzymatic colorimetric analysis with a fully automated autoanalyzer for clinical chemistry (Cobas 6000/c501; Roche Diagnostics GmbH, Vienna, Austria), as described recently [21].

### 2.4. Milk Data

Milk yields were recorded and milk samples were collected from the morning and afternoon milking of cows starting from 45 d postpartum (*i.e.*, on average four weeks after the surgery) during at least three occasions in a week interval. Milk samples of healthy cows were collected at similar time postpartum with DA cows. Milk samples were analyzed for fat, protein, and somatic cell counts (SCC) by Milkoscan (Foss Electric, Hillerød, Denmark).

### 2.5. Statistical Analyses

Data were first analyzed for normality using Shapiro-Wilk test. Except for NEFA and NEFA/Chol ratio, all other variables were normally distributed by this test (*p* < 0.05). Therefore, NEFA and NEFA/Chol were log-transformed for the parametric analysis. The analysis of variance was done using the MIXED procedure of SAS. An overall model was fitted to take into consideration the fixed effect of lactation number group (*i.e.*, ≤2 lactations and >2 lactations), health status, and their two-way interaction as well as the random effect of farm. The general statistical model was as follows:

(1) Yijk = μ + Pi + Hj + Fk + (PH)ij + eijk

in which Yijk represents the observation (*i.e.*, continuous variable such as serum variables or milk data) on cow k at lactation number group i of health status j and farm k; μ is the overall mean, Pi represents the fixed effect of the i-th lactation number group, i = 1, 2; Hj represents the fixed effect of the j-th health status, j = 1, 2; (PH)ij is the interaction between lactation number group and health status, and Fk represents the random effect of the k-th farm, k = 1, 2, … 14, and e the experimental error [22].

Degrees of freedom were approximated by the method of Kenward-Roger (ddfm = kr). Least square mean (LSM) and the respective standard error of the mean (SEM) were computed. Significance was declared at *p* ≤ 0.05, while a tendency was considered at 0.05 < *p* ≤ 0.10. To evaluate the effect of cow’s milk yield on metabolic variables, linear regression analyses using PROC REG of SAS were performed, as follows:

(2) Y = α0 + βX + e

in which *Y* represents the response variable; α*_0_* is the intercept, β is the regression slope and *X* describes the milk yield of cows, defined as explanatory variable, and *e* states for residuals. Only significant and tendentially significant (*p* < 0.10) relationships were considered. *p*-value and R^2^ were computed and used to evaluate the goodness of fit. All statistical analyses were carried out with SAS software (SAS Institute Inc., Cary, NC, USA, version 9.2).

## 3. Results

### 3.1. Serum Variables 

Data showed major alterations in several serum metabolic variables in cows affected by LDA (Table 1). These cows had dramatic increases of NEFA, NEFA/cholesterol ratio, and BHBA concentrations compared to healthy cows (*p* < 0.001). Aside from the health status, lactation number of the cows affected several serum variables. Accordingly, cows with ≤2 lactations had lower serum NEFA and BHBA concentrations of 25% and 50% respectively than cows with >2 lactations did (*p* < 0.05). Serum BUN was lower in cows with ≤2 lactation compared to cows >2 lactations (*p* < 0.05), and there was an interaction between the two factors on this variable (*p* = 0.077). Serum cholesterol was affected neither by LDA nor by lactation number.

**Table 1:** Serum variables of cows with left abomasal displacement (LDA) or healthy cows (HC) differing in lactation number.

Serum Variables ^1^	≤2 Lactations		>2 Lactations		SEM ^2^	*p*-value ^3^		
	LDA	HC	LDA	HC		Lact	Status	Int
Metabolites								
Cholesterol (mg/dL)	88.2	91.2	84.5	98.4	8.40	0.873	0.451	0.625
NEFA (mmol/L)	1.17	0.52	1.56	0.69	0.095	0.043	<0.001	0.434
BHBA (mmol/L)	2.11	0.74	4.67	1.00	0.428	0.028	<0.001	0.072
NEFA/cholesterol	0.018	0.006	0.023	0.007	0.002	0.258	<0.001	0.402
BUN (mg/dL)	23.7	35.2	35.6	35.9	2.23	0.046	0.981	0.077
Minerals								
Calcium (mmol/L)	1.92	2.21	1.89	2.42	0.035	0.093	<0.001	0.033
P (mmol/L)	1.86	1.77	1.85	1.67	0.102	0.651	0.294	0.713
Ca/P ratio	1.08	1.26	1.12	1.51	0.066	0.115	0.002	0.217
Liver enzymes								
AST (U/L)	147	74.9	450	84.2	68.2	0.126	0.034	0.150
GLDH (U/L)	41.8	9.9	73.6	16.7	14.1	0.240	0.009	0.445
GGT (U/L)	26.5	21.1	58.0	20.3	8.49	0.061	0.011	0.053
Acute phase proteins								
Hp (μg/mL)	2185	1212	1248	353	374	0.106	0.093	0.942
SAA (μg/mL)	126	59.1	132	60.0	22.2	0.904	0.033	0.927

**^1^** NEFA = non-esterified fatty acids; BHBA = beta-hydroxy-butyrate; BUN, blood urea nitrogen; AST, Aspartataminotransferase; GLDH, Glutamatdehydrogenase; GGT, Gamma-glutamyltransferase; Hp, Haptoglobin; SAA, Serum amyloid A; **^2^** SEM = standard error of the mean; **^3^** Effects of lactation number: Lact, cows were grouped in ≤2 lactations (*n* = 10 in LDA group and *n* = 11 in HC group) or >2 lactations (*n* = 10 in LDA group and *n* = 9 in HC group); Status, health status of cows; Int, Lact × Status interaction.

Serum minerals were not affected by lactation number, but LDA cows had reduced serum Ca concentrations compared to healthy cows (*p* < 0.001). There was an interaction between lactation number and serum Ca (*p* < 0.05), indicating a greater difference in Ca levels between sick and healthy cows with increasing lactation number. Likewise, the Ca/P ratio was lower in LDA cows (*p* < 0.05).

Serum activities of liver enzymes AST, GGT, and GLDH were significantly raised in cows with LDA compared to healthy cows (*p* < 0.05). However, there was no difference regarding the effects of lactation number on these enzymes, except of GGT values being greater in the sick cows with >2 lactations (*p* = 0.05).

Serum concentrations of Hp tended to be greater in cows with LDA (*p* = 0.09), and these cows also displayed elevated SAA concentrations compared to their healthy counterparts (*p* < 0.05). Lactation number and the interaction between this variable and health status showed no effect on both serum APP.

### 3.2. Milk Production and Composition 

Milk parameters measured four weeks after surgery differed between healthy cows and cows with LDA (Table 2). Milk protein tended to be lower whereas the fat/protein ratio and SCC tended to be greater in cows with LDA compared to healthy cows. Additionally, an interaction between lactation number and health status was observed for milk protein content (*p* < 0.05) suggesting that the percentage of milk protein was decreased by LDA in cows >2 lactations, but not in cows with ≤2 lactations. The same interaction was observed for milk fat percentage too. Overall, cows’ lactation number, affected several milk parameters, including milk yield, energy-corrected milk, fat and protein. Specifically, cows with >2 lactations had higher daily milk yield and protein yield (*p* < 0.001) than cows with ≤2 lactations. With increasing lactation number, milk fat and protein contents decreased (*p* < 0.05), whereas the daily milk fat yield increased (*p* < 0.05).

**Table 2:** Milk yield and composition in cows with left abomasal displacement (LDA) or healthy cows (HC) differing in the lactation number.

Milk Variables ^1^	≤2 Lactations		>2 Lactations		SEM ^2^	*p*-value ^3^		
	LDA	HC	LDA	HC		Lact	Status	Int
Milk yield (kg/d)	26.4	27.6	33.4	36.2	1.99	<0.001	0.360	0.708
ECM (kg/d)	27.6	26.1	29.6	34.7	1.96	0.013	0.384	0.114
Fat (%)	4.49	3.60	3.36	3.73	0.22	0.038	0.281	0.010
Protein (%)	3.27	3.22	2.92	3.22	0.08	0.041	0.144	0.043
Fat yield (kg/d)	1.16	0.99	1.10	1.34	0.09	0.146	0.734	0.049
Protein yield (kg/d)	0.85	0.88	0.97	1.16	0.05	0.002	0.067	0.195
Fat/protein ratio	1.37	1.12	1.16	1.15	0.06	0.188	0.062	0.071
SCC (cells/mL)	141.8	79.5	379.8	104.2	66.7	0.173	0.082	0.266

**^1^** Milk data were recorded 3 times in a week interval starting from 45 d postpartum (almost four weeks after the surgery); ECM, energy-corrected milk = (kg milk × (0.383 × % fat + 0.242 × % protein + 0.7832)/3.1138); SCC, somatic cell count (expressed in × 1000 cells/mL); **^2^** SEM = standard error of the mean; **^3^** Effects of lactation number: Lact, cows were grouped in ≤2 lactations (*n* = 10 in LDA group and *n* = 11 in HC group) or >2 lactations (*n* = 10 in LDA group and *n* = 9 in HC group); Status, health status of cows; Int, Lact × Status interaction.

### 3.3. Associations between Milk Yield and Serum Variables 

**Figure 1:**
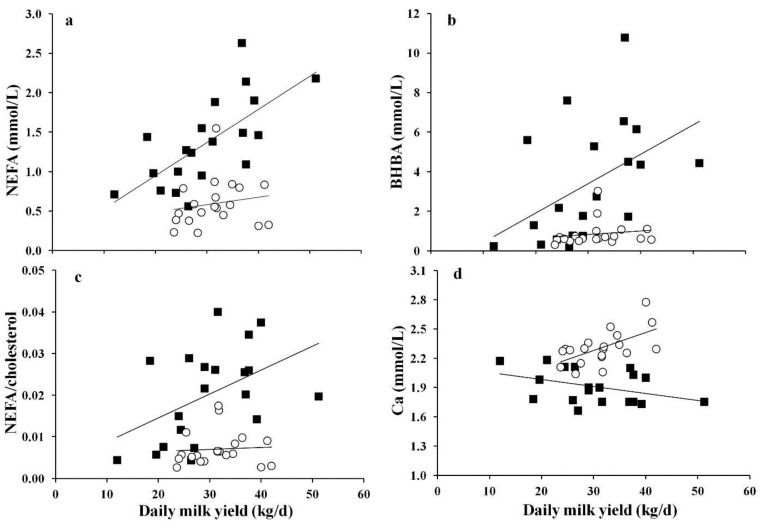
Association between milk yield and serum (**a**) non-esterified fatty acids (NEFA); (**b**) beta-hydroxy-butyrate (BHBA); (**c**) NEFA/cholesterol ratio; and (**d**) calcium in cows affected by left abomasal displacement (■) or not (○). Significant associations in cows affected with left abomasal displacement: NEFA = 0.11 + 0.420 × milk yield, R^2^ = 0.45, *p* < 0.001; BHBA = −1.06 + 0.149 × milk yield, R^2^ = 0.21, *p* = 0.039; NEFA: cholesterol = 0.008 + 0.00025 × milk yield, R^2^ = 0.22, *p* = 0.035; Ca = 2.13 − 0.0072 × milk yield, R^2^ = 0.11, *p* = 0.094. Significant associations in healthy cows: Ca = 1.72 + 0.018 × milk yield, R^2^ = 0.35, *p* = 0.006.

Associations between milk yield data and blood variables are shown in Figure 1. Cows with LDA showed a linear increase in serum NEFA, BHBA, as well as NEFA/cholesterol ratio with increasing milk yield (R^2^ = 0.21 to 0.45, *p* < 0.05), albeit not showing any significant relationship in healthy cows (Figure 1a–c). Moreover, milk yield was positively linearly related to serum Ca (Figure 1d) as well as Ca:P ratio (data not shown) in healthy cows (R^2^ = 0.35 to 0.42, *p* < 0.01), whereas an opposite effect was observed for the association between serum Ca and daily milk yield in cows affected by LDA (R^2^ = 0.11, *p* = 0.094, Figure 1d).

## 4. Discussion

### 4.1. Metabolic and Inflammatory Responses

This research aimed to investigate metabolic and inflammatory responses associated with LDA in high-producing dairy cows kept under small-scale farm conditions. We observed dramatic increases of NEFA and BHBA concentrations associated with higher SAA in the sick cows, which are typically findings reported for LDA cows [1,5,13]. High concentrations of serum BHBA derive from the oxidation of excessive NEFA in hepatocytes due to an enhanced lipolysis and the lack of energy, which is aggravated in LDA cows because of excessive NEB [1]. A NEB in postparturient dairy cows with LDA has been reported to lead not only to an excessive lipolysis but also to the release of proinflammatory cytokines such as TNF-α from adipocytes, resulting both in elevated serum NEFA and APR conditions with increased hepatic synthesis of SAA [14]. This assumption is supported by our findings of greater NEFA and NEFA/cholesterol, as well as SAA levels in the LDA cows. The elevated ratio of NEFA/cholesterol is indicative of fat infiltration of the liver [23], whereas greater SAA suggest an inflammatory condition in LDA cows [12]. Furthermore, the tendency for higher milk fat/protein ratio and lower protein yield in LDA cows is indicative of a more severe energy deficiency in association with greater body mass losses, which are known to contribute to increasing milk fat/protein ratio [24].

Typically, all cows in the immediate postparturient period experience an energy and nutrient deficit, whereby cows experiencing LDA lower their feed intake long before the occurrence of disease [1]. The latter event aggravates further the energy and nutrient deficit immediately after parturition. This causal relationship likely explains the current findings of dramatic increase of NEFA and BHBA concentrations in the serum of cows with LDA. Several authors claimed serum BHBA concentrations around 1.6 mmol/L in the first week post-partum as BHBA cut-point for prediction of LDA risk [5,25,26]. In the present study, blood samples were collected at the time point of surgery and most of the cows, in particular those with high milk yields were already in a clinical ketotic state, showing about three times higher BHBA values than healthy cows.

In agreement with the present results, elevated serum levels of the enzyme AST have been reported in cows with LDA in previous studies [13,27]. We also observed significant increases in serum GGT and GLDH, which may indicate liver cell damage in LDA cows [13], possibly caused by hepatic lipidosis. In addition, a rise of blood AST activity has been assumed to result from muscle protein mobilization or depletion aiming to deliver glycogenic amino acids as glucose precursors [28], which was supported by the detected lower concentration of milk protein in LDA cows.

Although serum Hp only showed a tendency, the data of SAA support the line of thinking that development of LDA is associated with an inflammatory state. This assumption has been supported by several other reports [12,13,14,15]. The fact that Hp did not significantly differ between groups can likely be explained by the fact that this marker is more involved in the subacute or chronic inflammatory conditions rather than acute inflammation processes in cattle [29]. Despite little evidence linking development of systemic APR to lowering levels of blood Ca [14,15] and also a relationship between low serum Ca and LDA occurrence [15,26,30,31], the mechanisms by which APR is linked to the development of LDA has not yet been established. It is generally accepted that low blood Ca inhibits abomasal motility [9]. Supporting the theory of a disturbed Ca homeostasis, cows with LDA had a decreased serum Ca concentration compared to healthy cows in the present study. The serum concentration of LDA cows was <2 mmol/L, a concentration which has been reported to increase the risk of LDA [31].

### 4.2. Effects of Lactation Number and Milk Yield on Blood Variables

An important aim of this study was to establish an association between lactation number and milk production with the outcome of metabolic and inflammatory variables. Regarding the effect of lactation number, the present data demonstrated that cows in the third or higher lactation showed higher serum values of NEFA, BHBA, NEFA/cholesterol ratio, and GGT than cows in the first or second lactation. These results led to the assumption that higher lactation numbers exacerbate metabolic alterations associated with LDA likely due to higher milk yields. Indeed, interesting findings of the present study were the association between increasing milk yield and reduced serum Ca, and increasing NEFA, BHBA, and NEFA/cholesterol ratio in LDA cows. The reasons why milk yield only exacerbates metabolic variables, but not inflammatory and liver enzymes in LDA cows, and not in healthy cows, is not known and requires further investigation. It is possible that high milk production leads to greater drainage of nutrients via milk in LDA cows, which cannot be compensated by nutrient intake. It is also likely that high milk production aggravates the catabolic state provoked by LDA, leading to exacerbation of metabolic alterations, which were not observed in healthy cows. It seems that other factors besides milk production are involved in these strong metabolic alterations. Already Martin [32] pointed out that, due to strong metabolic and endocrine changes, parturition could predispose all cows to LDA; however, only certain individuals have other important predisposing factors which favor the development of LDA [33].

A NEB is well-known as the initiating factor in the development of both ketosis and hepatic lipidosis, which have been implicated as risk factors for LDA [8]. The present data indicate that in cows affected by LDA a high daily milk yield results in a deeper or more long-lasting NEB around calving. Oppositely, healthy cows appear to be in less severe NEB, even at comparably higher milk yield levels, which indicates a better adaptation of these cows during the initiation of the lactation.

Interestingly, in healthy cows, serum Ca as well as Ca/P ratio increased with increasing milk yield, whereas the opposite effect was observed for Ca in LDA cows. Despite assumed higher feed intake in healthy cows, this might be related to a higher ability of healthy cows to respond to the developing hypocalcemia by secreting parathyroid hormone, enabling Ca mobilization from bone, as well as enhanced renal reabsorption and efficient intestinal absorption [34]. In contrast, these homeostatic mechanisms appear to fail to operate sufficiently in cows affected by LDA, resulting in hypocalcemic conditions, becoming even more pronounced with increasing milk yield. Thus, the obtained results let assume that increasing milk yield impairs Ca metabolic regulation mechanisms in cows with LDA, whereas this is not the case in healthy cows.

The comparison among groups of cows regarding serum cholesterol should be interpreted with caution, because cows were not matched by lactation day. Serum cholesterol is a variable which is very sensitive to lactation day in cows [2]. Another limitation of this study is the disparity of lactation number of the cows in the LDA and HC group, in a way that eight LDA cows were of four lactations and higher, whereas only two cows in the HC group had this number of lactations. Studies with equal number of lactations between groups are needed to verify the present findings.

## 5. Conclusions 

Taken together, the study indicated metabolic alterations, liver damage, and inflammation in LDA cows kept under small-scale farm conditions. Our data also suggest exacerbation of metabolic profile and Ca metabolism but not of inflammation and liver health with increasing lactation number and milk yield in LDA cows, and not in healthy cows. From a clinical point of view, the current data suggest the need of a rapid intervention to re-establish the aggravated ketonemia and hypocalcemia, liver health, and inflammation, whereby high-producing LDA cows with >2 lactations require more attention regarding metabolic variables than younger cows.
